# 128 Prolonged KPC-CRE Outbreak with Horizontal Plasmid Transfer Associated with ICU Sink Drains

**DOI:** 10.1017/ash.2026.10540

**Published:** 2026-06-23

**Authors:** Natalie Stanley, Sarah Petersen, Malakai Miller, Samantha Mathieson, Simone Godwin

**Affiliations:** 1 Tennessee Department of Health; 2 TN Department of Health

## Abstract

**Background:** Clostridioides difficile is one of the most common healthcare associated infections. Due to diagnostic uncertainty, patients are often treated for colonization instead of infection. Colonization is not believed to be a direct precursor for a Clostridioides difficile infection (CDI) and does not require treatment. The aim of this study is to analyze factors associated with overtreatment for Clostridioides difficile colonization, and whether these factors contribute to recurrence. **Methods:** This study examined fully abstracted incident Clostridioides difficile cases, defined as the first positive stool test for persons at least one year of age residing in Davidson County, Tennessee from 2018–2022. Colonization was determined by negative enzyme immunoassay test combined with positive polymerase chain reaction test. Overtreatment was defined as receiving treatment for colonization. Recurrence was defined as a positive stool specimen between two to eight weeks of the last positive specimen. Overtreatment and recurrence were analyzed with two separate logistic regression models using SAS 9.4. **Results:** There were 1,097 patients examined. Of these patients, 61% were overtreated for colonization. Patients without comorbidities and with increasing age had lower odds of overtreatment (OR=0.6, 95% CI [0.4–0.9]; OR=0.7, 95% CI [0.6–0.8]). Patients with hospitalizations on the day of or in the six calendar days after the specimen collection or with diverticulitis or inflammatory bowel disease were significant for higher odds of overtreatment (OR=1.3, 95% CI [1.0–1.8]; OR=1.6, 95% CI [1.1–2.4]; OR=2.1, 95% CI [1.2–3.8]). There was no significance found for predicting overtreatment with other demographics or comorbidities (e.g., diabetes mellitus, chronic kidney disease, chronic pulmonary disease). Increasing age had higher odds of recurrence (OR=1.3, 95% CI [1.0–1.6]). No other factors associated with overtreatment were associated with recurrence. **Conclusion:** Overtreatment was common in this population. Hospitalization contributed to increased overtreatment of Clostridioides difficile colonization, and it is important to consider patient comorbidities that may present similarly to clinical CDI when considering treatment. Future studies should examine how overtreatment can affect other CDI outcomes and additional evaluation of general prevalence in the population, incidence of CDI, and mortality rates following overtreatment.

